# Emulation of Astrocyte Induced Neural Phase Synchrony in Spin-Orbit Torque Oscillator Neurons

**DOI:** 10.3389/fnins.2021.699632

**Published:** 2021-10-12

**Authors:** Umang Garg, Kezhou Yang, Abhronil Sengupta

**Affiliations:** ^1^School of Electrical Engineering and Computer Science, Department of Materials Science and Engineering, The Pennsylvania State University, University Park, PA, United States; ^2^Department of Electronics and Instrumentation Engineering, Birla Institute of Technology and Science, Pilani, India

**Keywords:** neuromorphic computing, magnetic tunnel junction, astrocytes, Spintronics, spiking neural networks

## Abstract

Astrocytes play a central role in inducing concerted phase synchronized neural-wave patterns inside the brain. In this article, we demonstrate that injected radio-frequency signal in underlying heavy metal layer of spin-orbit torque oscillator neurons mimic the neuron phase synchronization effect realized by glial cells. Potential application of such phase coupling effects is illustrated in the context of a temporal “binding problem.” We also present the design of a coupled neuron-synapse-astrocyte network enabled by compact neuromimetic devices by combining the concepts of local spike-timing dependent plasticity and astrocyte induced neural phase synchrony.

## 1. Introduction

Neuromorphic engineering is emerging to be a disruptive computing paradigm in recent times driven by the unparalleled efficiency of the brain at solving cognitive tasks. Brain-inspired computing attempts to emulate various aspects of the brain's processing capability ranging from synaptic plasticity mechanisms, neural spiking behavior to *in-situ* memory storage in the underlying hardware substrate and architecture. The work presented in this article is guided by the observation that current neuromorphic computing architectures have mainly focused on emulation of bio-plausible computational models for neuron and synapse—but have not focused on other computational units of the biological brain that might contribute to cognition.

Over the past few years, there has been increasing evidence that glial cells, and in particular, astrocytes play an important role in multitude of brain functions (Allam et al., 2012). It is estimated that glia form ~50% of the human brain cells (Möller et al., 2007) and participate by modulating the neuronal firing behavior, though unable to discharge electrical impulses of their own. Indeed, these glial-cells work in coordination with neural assemblies, to enable information processing in the human brain and performing incisive operations. Astrocytes hold the recipe to potentiate or suppress neurotransmitter activity within networks and are responsible for phenomenon like synchronous network firing (Fell and Axmacher, 2011; Wade et al., 2011) and self-repair mechanisms (Wade et al., 2012; Rastogi et al., 2020). It is therefore increasingly important to capture the dynamics of such ensembles, a step toward realizing more sophisticated neuromimetic machines and ultimately enabling cognitive electronics.

Recently, there has been extensive literature reporting astrocyte computational models and their impact on synaptic learning (De Pittà et al., 2012; Manninen et al., 2018). Continuing these fundamental investigations to decode neuro-glia interaction, there have been recent neuromorphic implementations of astrocyte functionality in analog and digital Complementary Metal Oxide Semiconductor (CMOS) hardware (Möller et al., 2007; Irizarry-Valle and Parker, 2015; Naeem et al., 2015; Ranjbar and Amiri, 2017; Karimi et al., 2018; Faramarzi et al., 2019). For instance, analog CMOS circuits capturing the neural-glial transmitter behavior have been demonstrated (Joshi et al., 2011; Irizarry-Valle et al., 2013; Irizarry-Valle and Parker, 2015; Lee and Parker, 2016). There is also increasing interest in low-complexity FPGA implementation of the astrocyte computation models (Nazari et al., 2015; Ranjbar and Amiri, 2016, 2017; Karimi et al., 2018; Faramarzi et al., 2019). However, the primary focus has been on a brain-emulation perspective, i.e., implementing astrocyte computational models with high degree of detail in the underlying hardware.

On the other hand, recent advances in emerging post-CMOS technologies like phase change materials, resistive memories, ferromagentic, and ferroelectric materials (Jo et al., 2010; Kuzum et al., 2011; Ramakrishnan et al., 2011; Jackson et al., 2013; Sengupta and Roy, 2017; Saha et al., 2021), among others have resulted in the development of electronic device structures that can reproduce various biomimetic characteristics at low operating voltages through their intrinsic physics. However, while there has been extensive work on exploring post-CMOS technologies for mimicking bio-realistic computations due to the prospects of low-power and compact hardware design, they have been only studied from standalone neuron/synapse perspective. Emulation of the neuron-astrocyte crosstalk using bio-mimetic devices has largely been neglected, and no such literature exists hitherto, to the best of our knowledge. This work is therefore an effort to bridge this gap and, specifically, elucidates the emulation of transient synchronous activity resulting from neural-glial interactions by utilizing spin-orbit torque induced phase synchronization of spintronic oscillator neurons. It is worth mentioning here that we abstract the neuron functionality as a non-linear oscillator, in agreement with prior neuroscience and computational models (Jaeger and Haas, 2004). Emulation of astrocyte induced neural phase synchrony through the intrinsic physics of spintronic devices will be critical to enable the next generation of resource constrained cognitive intelligence platforms like robotic locomotion (Polykretis et al., 2020). This work also presents an important addition to the wide variety of next-generation computational paradigms like associative computing, vowel-recognition, physical reservoir computing among others (Fan et al., 2015; Torrejon et al., 2017; Romera et al., 2018, 2020; Riou et al., 2019; Tsunegi et al., 2019), being implemented using spin-torque oscillator devices.

## 2. Neuroscience Background

**Figure 1:**
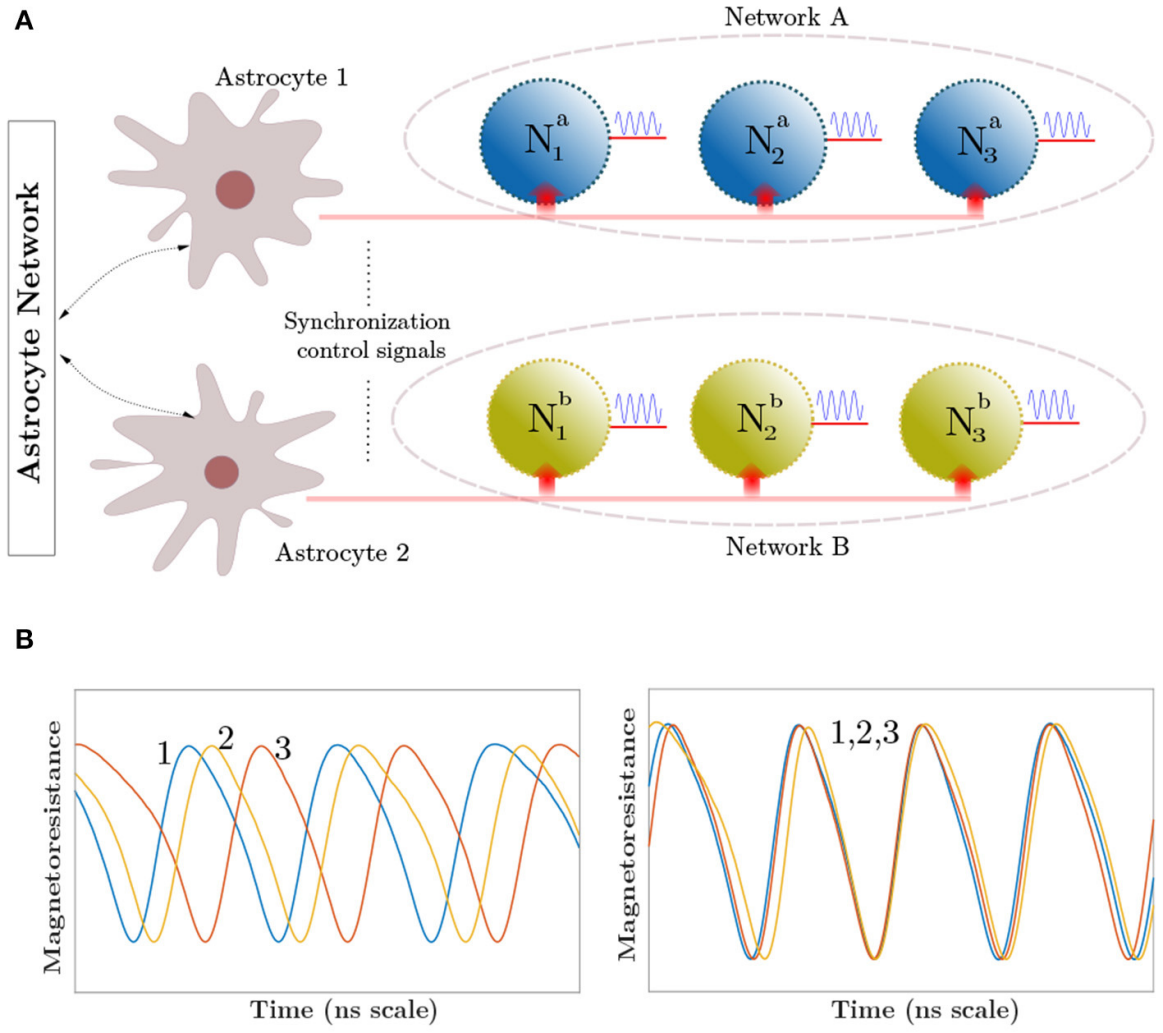
**(A)** Top-level network depicting the synchronization control by astrocytic injection. Astrocytes share information among their glial network. **(B)** The curves show the synchronized and unsynchronized outputs of Neurons 1–3 in Network A depending on the astrocyte input.

The human brain houses multiple-independent local neuronal groups which perform dedicated computations in relevance to their assigned tasks. Besides this general uncorrelated activity of neurons, multiple neural spiking data recordings reveal that the independent signals from these neural assemblies frequently coalesce in time to generate a synchronous output (Fries, 2005; Fell and Axmacher, 2011). Multiple reports on the cause of such patterns now provide compelling evidence that astrocytes are the agents of this phenomenon (Fellin et al., 2004; Wade et al., 2011). Astrocytes modulate the concentration of neurotransmitters like glutamate inside the synaptic clefts in response to its internal Calcium (*Ca*^2+^) oscillations (Newman, 2003; Garbo et al., 2007). A single astrocyte spans tens of thousands of synapses, where units called microdomains (concentrated *Ca*^2+^ stores within the astrocyte) monitor the activity for a group of neurons and perform subsequent chemical actions (Volterra and Meldolesi, 2005; Haydon and Carmignoto, 2006). The astrocyte-derived glutamate binds to extrasynaptic NMDAR (N-methyl-D-aspartate) receptor channels, and induce Slow-inward Currents (SIC) in the post-synaptic membrane. SICs are attributed to triggering a simultaneous response in different synapses with high timing precision, and its large amplitude and slow-decay rate provide an increased timescale for the correlated activity (Fellin et al., 2004; Wade et al., 2011). The astrocytic units influencing synapses, can act both independently or in coordination enabling long-distance indirect signaling among independent neuronal groups. Furthermore, an increased intensity of synaptic activity can trigger multiple astrocytes to share their information through their gap-junctions and elicit coherent behaviors among different uncorrelated neuronal networks. We in this paper do not discriminate among the two signaling processes. Thus, the two astrocytes shown in Figure 1A for different sub-networks can also imply microdomains within a single astrocyte. These units control the synchronization signal to networks A and B. Figure 1A captures the biological perspective of such a system which controls the neural synchronization among neurons present in these different sub-networks. Sub-networks A and B each consist of three different neurons, which in-turn generate oscillatory outputs. The temporal profiles, shown in Figure 1B, depict the neuron outputs before and after synchronization is initiated by Astrocyte 1 in the network A. Interested readers are referred to Wade et al. (2011) for details on the astrocyte computational models. It is worth mentioning here that unlike CMOS implementations that are able to implement computational models with a high degree of detail, emerging device based implementations usually focus on mimicking key aspects of the neurosynaptic functionality necessary from computing perspective since the exact behavior is governed by the intrinsic device physics. In this work, we primarily consider emulating the neural phase synchrony effect of astrocytes and evaluate it in the context of a temporal information binding application.

## 3. Astrocytic Synchronization Emulation

### 3.1. Device Basics

In this work, we utilize Magnetic Tunnel Junctions (MTJs) (Julliere, 1975) as the core hardware primitive to mimic neural oscillations. The MTJ consists of two ferromagnetic layers (pinned layer and free layer) with a spacer oxide layer in between. The direction of magnetization of the pinned layer (PL) is fixed, while that of the free layer (FL) can be manipulated by external stimuli (spin current/magnetic field). The MTJ stack exhibits a varying resistance depending on the relative magnetic orientations of the PL and the FL. The extreme resistive states are referred to as the parallel (P) and anti-parallel (AP) states depending on the relative FL magnetization. The magnetization dynamics of the FL can be modeled by Landau-Lifshitz-Gilbert-Slonczewski (LLGS) equation with stochastic thermal noise (Sengupta and Roy, 2017):


(1) $$\frac{d\hat{m}}{dt}=-\gamma (\hat{m}\times H_{eff})+\alpha (\hat{m}\times \frac{d\hat{m}}{dt})+\frac{1}{qN_{s}}(\hat{m}\times I_{s}\times \hat{m})$$

In Equation (1), $\hat{m}$ is the unit vector representing the magnetization direction of FL, *H*_*eff*_ is the effective magnetic field including thermal noise (Scholz et al., 2001), demagnetization field and external magnetic field, γ is the gyromagnetic ratio, α is Gilbert's damping ratio, *I*_*s*_ is the spin current, *q* is the electronic charge, and $N_{s}=\frac{M_{s}V}{\mu_{B}}$ is the number of spins in free layer of volume *V* (*M*_*s*_ is saturation magnetization and μ_*B*_ is Bohr magneton). If the magnitude of spin current and external magnetic field are chosen appropriately such that the damping due to the effective magnetic field is compensated, a steady procession of the FL magnetization can be obtained. It is worth mentioning here that the intrinsic magnetization dynamics in Equation (1) is used to model the oscillator dynamics. Other variants of oscillatory behavior can be achieved by modified spin device structures (Matsumoto et al., 2019).

**Figure 2:**
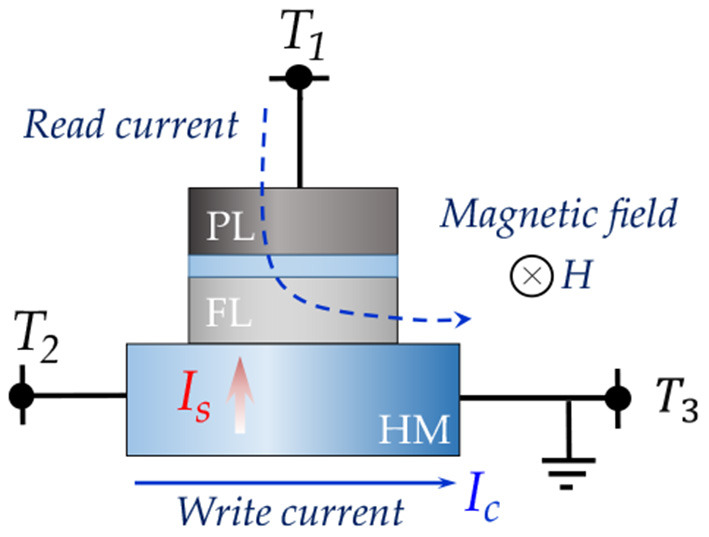
Spin-orbit torque device undergoes oscillation due to applied external magnetic field, *H*, and charge current, *I*_*c*_. Note that the directions of both the magnetic field and magnetic anisotropy are in-plane.

In order to achieve decoupled output oscillator readout and astrocyte injection induced phase coupling, we utilize a three terminal device structure, as shown in Figure 2, in which a nanomagnet with in-plane magnetic anisotropy lies on top of a heavy metal (HM) layer with high spin-orbit coupling. Due to spin-Hall effect (Hirsch, 1999), a transverse spin current is injected into the MTJ FL by charge current, *I*_*c*_, flowing through the HM between terminals T2 and T3. The relation between spin current *I*_*s*_ and charge current *I*_*c*_ is,


(2) $$I_{s}=\theta_{SH}\frac{A_{FM}}{A_{HM}}\left(1-\text{sech}\left(\frac{t_{HM}}{\lambda_{sf}}\right)\right)I_{c}$$

where, *A*_*FM*_ and *A*_*HM*_ are the FM and HM cross-sectional areas respectively, θ_*SH*_ is the spin-Hall angle (Hirsch, 1999), *t*_*HM*_ is the HM thickness and λ_*sf*_ is the spin-flip length. Note that an in-plane magnetic field, *H*, is also applied to achieve sustained oscillation. The MTJ state is read using the current sensed through terminal T1. The device simulation parameters are tabulated in Table 1 and are based on typical experimental measurements reported in literature (Fan et al., 2015). However, the conclusions presented in this study are not specific to these parameters. Experimental demonstration of injection locked spin-torque oscillators have been achieved (Rippard et al., 2005, 2013; Georges et al., 2008; Demidov et al., 2014). It is worth mentioning here that we assume all the devices are magnetically isolated and sufficiently spaced such that dipolar coupling is negligible (Yogendra et al., 2017). We also consider that the generated charge current in the HM layer due to FL magnetic precession via the Inverse spin-Hall effect (ISHE) is not dominant enough to impact the phase coupling phenomena. While recent studies have shown that the ISHE modulated current alone, without any amplification, is not sufficient to impact phase locking (Elyasi et al., 2015), such effects can be also overcome by limiting the number of oscillators sharing a common HM substrate.

**Table 1:** MTJ device simulation parameters.

**Parameters**	**Value**
Ferromagnet area, *A*_*FM*_	40 ×100 nm
HM thickness, *t*_*HM*_	3 nm
Energy barrier, *E*_*b*_	62.76 kT
Saturation magnetization, *M*_*s*_	$\frac{10^{7}}{4\pi }$ A/m
Spin-hall angle, θ_*SH*_	0.3
Spin-flip length, λ_*sf*_	1.4 nm
Gilbert damping factor, α	0.03
External magnetic field, *H*	750 Oe
TMR ratio, *TMR*	200%
Temperature, *T*	300 K

### 3.2. Phase Synchronization of MTJ Oscillator Neurons

**Figure 3:**
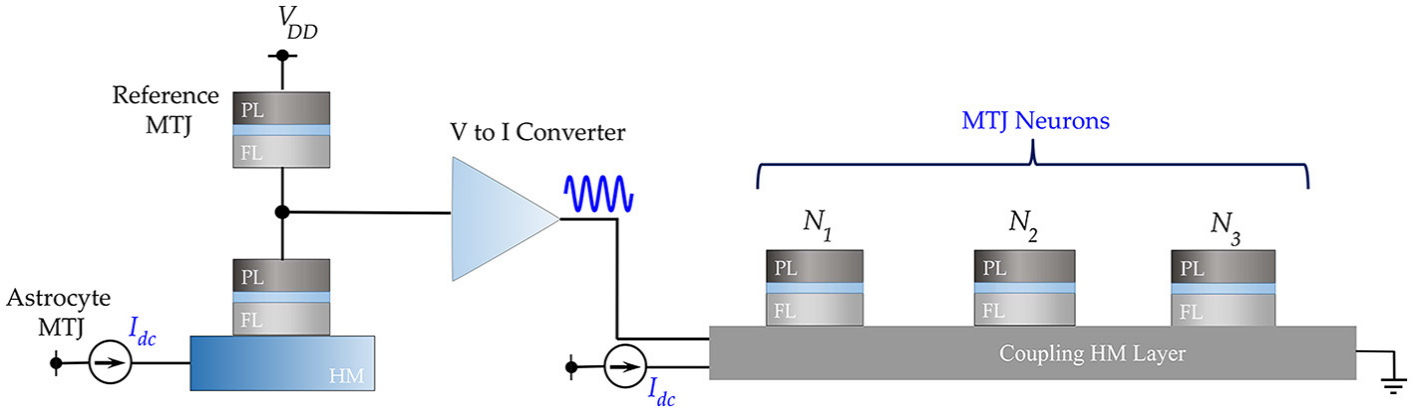
Electrical emulation of astrocyte induced neural synchrony is shown where an astrocyte device drives an alternating current through a common HM substrate to phase-lock the MTJ oscillator neurons.

**Figure 4:**
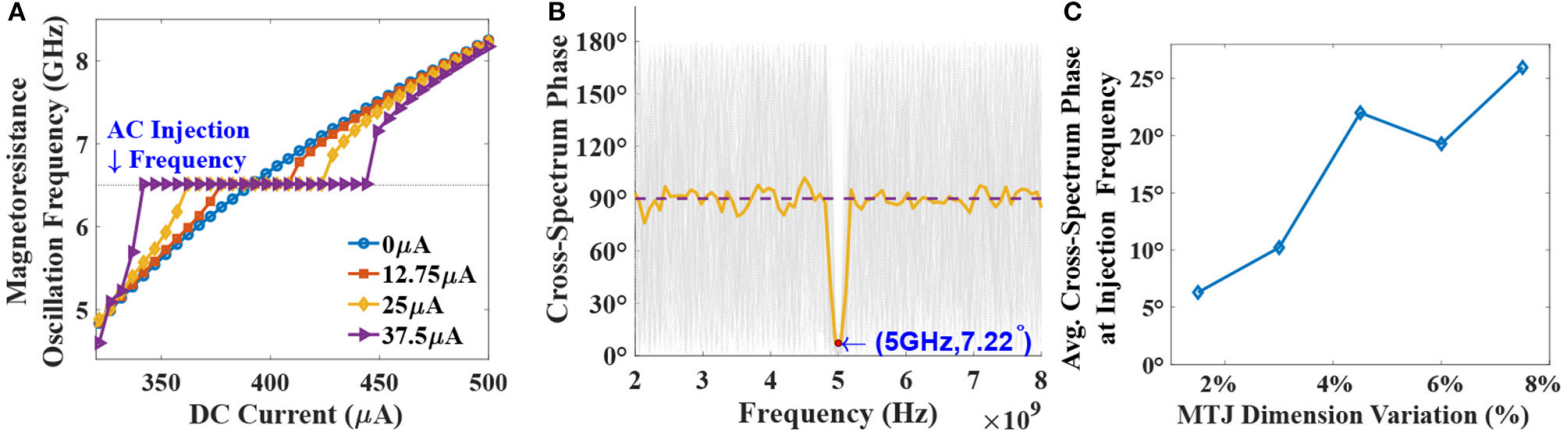
**(A)** Oscillator frequency plotted against the DC current input to the device. Higher AC amplitudes lead to increased DC locking range at the injected RF signal of 6.5 GHz frequency. **(B)** Cross-spectrum phase for 100 independent stochastic LLGS simulations of two noisy MTJ neurons, under RF injection of 5*GHz*. Average CPSD phase indicates tight phase-coupling at the required frequency with un-correlated activity at other frequencies. **(C)** Average cross-spectrum phase at the injection frequency accounting for device dimension variations.

The electrical analog of Figure 1A is shown in Figure 3, where the MTJs represent the oscillatory neurons present in a particular network. The neurons share a HM layer which acts as the common substrate for the driving astrocyte signal. The current flowing through the HM has two components—a DC current input which determines the free-running frequency of the oscillator and a radio-frequency signal which represents the astrocyte input. Figure 4A highlights the oscillation characteristics of the MTJ. The DC current controls the precession frequency in absence of other inputs. This DC input is analogous to the external stimulus determining the frequency of neuron oscillation in a particular network. In the absence of the RF signal, all the neurons oscillate at the same frequency (dependent on stimulus magnitude or DC current) but out-of-phase due to thermal noise. Upon the application of the external RF astrocyte signal, the device oscillation locks in phase and frequency to this input. Higher peak-to-peak amplitude of the astrocyte locking signal increases the locking range of the device. It is worth mentioning here that the locking frequency of neurons in a particular network is dependent on the stimulus and astrocytes only induce phase locking. Therefore, the alternating astrocyte signal flowing through the HM layer can be generated from a separate astrocyte device that is driven by the corresponding DC input of the network, thereby ensuring independent phase and frequency control. The astrocyte device is interfaced with a Reference MTJ and a voltage-to-current converter to drive the alternating current signal through the common HM layer. The Reference MTJ state is fixed to the AP state (by ensuring that the read supply voltage, *V*_*DD*_ = 0.65*V* is not high enough to write the MTJ state) and forms a resistive divider with the oscillating Astrocyte MTJ resistance. Therefore, the gate voltage of the interfaced PMOS transistor, $V_{G}=\frac{R_{A}}{R_{A}+R_{REF}}V_{DD}$ where *R*_*A*_ is the Astrocyte MTJ resistance and *R*_*REF*_ is the Reference MTJ resistance, also varies accordingly, which in turn, modulates the current flowing through the common HM layer proportionally.

In order to evaluate the degree of phase synchronization in presence of thermal noise, we consider two MTJ devices lying on top of a common HM layer at room temperature. Cross-correlation metric is evaluated for the two MTJ output signals to measure the similarity among them as a function of displacement of one relative to the other. Considering two time-domain functions *x*(*t*) and *y*(*t*), whose power spectrum density (PSD) is given by *S*_*xx*_(ω) and *S*_*yy*_(ω), respectively, their cross-correlation is defined by:


(3) $$R_{xy}(\tau )=(x⋆y)(\tau )=\int_{-\infty }^{\infty }\bar{x(t-\tau )}y(t)dt$$

where, $\bar{x\left(t\right)}$ represents the complex conjugate of *x*(*t*) and τ denotes the lag parameter. Further, cross-power spectral density (CPSD) is defined as the Fourier transformation of cross-spectrum in (3) and is given by:


(4) $$S_{xy}(\omega )=\int_{-\infty }^{\infty }R_{xy}(t)e^{-j\omega t}dt$$

*S*_*xy*_ comprises of both magnitude and phase (∠) information at different frequencies present in $\left[\begin{array}{c} \omega \end{array}\right]$ vector. When two signals are phase synchronized, the cross-spectrum phase vector becomes zero, indicating high correlation. Such a property is highlighted in Figure 4B where 100 independent stochastic-LLGS simulations are performed for two neuronal devices placed on a common HM layer with a 5 GHz injected RF current. Cross-spectrum phase at the injection frequency, i.e., 5 GHz converges close to zero. Average cross-spectrum phase is also shown in the plot depicting tight phase-coupling between the neurons at the injection frequency. Notably, a sharp reduction of average phase offset to just 7.22° at 5 GHz is observed compared to 90° for other frequencies, thereby establishing the robustness of the synchronization scheme. Additionally, the impact of non-idealities like device dimension variations on the phase coupling phenomena is evaluated in Figure 4C. The results are reported for 50 independent Monte-Carlo simulations with variation in both the length and width of the MTJ. Each Monte-Carlo simulation consisted of 50 stochastic LLGS simulation for the average cross-spectrum phase calculation. The phase correlation between the device oscillations remains reasonably high even with 7.5% variation in both length and width dimensions of the MTJ. Related discussions on oscillator dynamics with respect to perturbative current and correspondence of the results with the Kuramoto model for oscillator synchronization is provided in the Supplementary Material.

## 4. Binding Problem

### 4.1. Problem Formulation

**Figure 5:**
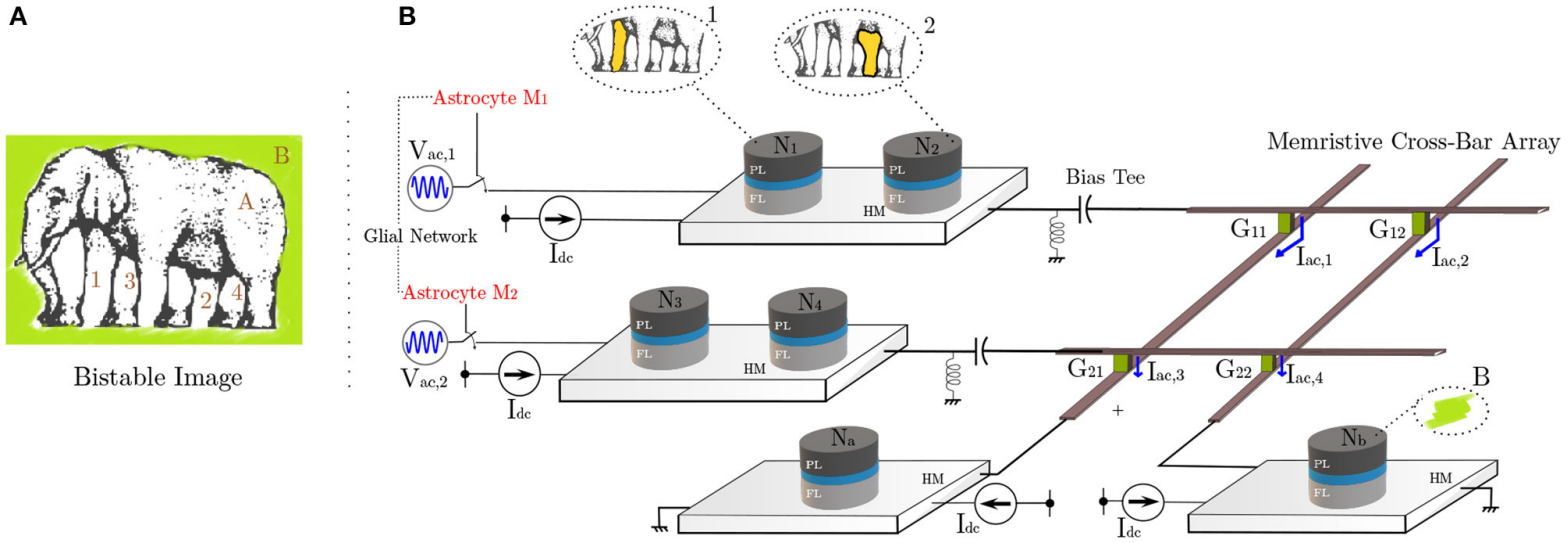
**(A)** The optical illusion induces confusion in the viewer concerning association among different apparent limbs with the body and the background (*Courtesy of Roger Shepard's “L'egsistential paradox”*) (Shepard, 1990). **(B)** MTJ system architecture depicting hierarchical organization of neurons. The illustrated binding problem is mapped to this hardware with one possible interpretation shown. The connection between different neuron layers is implemented by the memristive cross-bar array with initially untuned synaptic weights. Unsupervised STDP learning rule causes the weights to evolve, making the network to finally elicit synchronous responses post-training.

Next, we discuss a renowned problem which is envisioned to be solved by neural synchronous activity. Amongst the most intriguing themes of neuro-psychological studies is the “binding problem” (BP) (Feldman, 2013; Fields et al., 2014). It concerns with how different attributes of sensory information are encoded, processed, and perceived for decision-making by the human brain circuits. With a now widely accepted viewpoint of distributive computing and segregated processing for different features (especially visual) and later integration into a unified percept via re-entrant connections (Milner, 1974; Bartels and Zeki, 2006), we have progressed further toward understanding cognition. Primate brains have evolved to continuously assimilate the voluminous perceptive information available in their social setting and find a best fit for the primate's goals in the quickest manner. This training and growth, although very crucial in most situations—sometimes also leads to “misbinding” (Whitney, 2009). In particular, optical illusions, such as shown in Figure 5A, exploit the feature patterns ingrained in the human visual percept, causing misbinding. The figure is a bistable portrait of an elephant, or an overlap of two (seemingly) possible interpretations, obtained by associating different body parts to other features of the image. For instance, the labels 1 and 2 can be viewed associated with the body (A), while 3 and 4 to the background (B) to paint one such possible interpretation. The other interpretation can be visualized if the roles A and B are reversed. For an in-depth discussion, interested readers are directed to Hasz and Miller (2013) and Ignatov et al. (2017). In this work, we do not address the clustering mechanism of labels 1–2 and 3–4. This labeling and identification can be potentially attributed to the agent's visual attention. In particular, attention captures the most relevant information present in a space-time lapse by masking (filtering) off the distractor areas, while performing feature labeling of the cropped scene (Kosiorek et al., 2017). Assuming that attention performs the role of spatio-temporal integration among such multiple attributes captured by a visual scene, synchronous activity in the neurons is considered as the underlying mechanism in brain to create a coherent episode of perception, and perhaps cognition. Indeed, it is now becoming more evident that cognitive processes like attention and behavioral efficiency elicit targeted synchronous activity in different brain regions tuned to responding toward different spatial and featural attributes of the attended sensory input (Ward, 2003; Womelsdorf and Fries, 2007).

### 4.2. Hardware Mapping

In order to correlate our spin-orbit torque oscillator phase synchronization due to astrocyte injection locking in the context of “temporal binding,” we consider a network as shown in Figure 5B. Adhering to the currently prominent view of hierarchical organization in the neural assemblies, spin-torque neurons *N*_1_, *N*_2_, *N*_3_, *N*_4_ here are dedicated to processing simple attributes, while *N*_*a*_ and *N*_*b*_ after receiving inputs from previous layers perform complex feature processing corresponding to the assigned task. In reference to potential processing applications like cognitive feature binding, each spin-orbit torque neuron in the network represents the corresponding feature in the elephant's bistable image, previously shown in Figure 5A. All neuronal devices are mounted atop a HM with *I*_dc_ = 420μ*A* DC drive (*f*_free_ = 7.05 GHz). The network utilizes two different injection signals with the same frequency of 7.05 GHz with 180^*o*^ phase difference (corresponding to the two different interpretations/configurations of the bistable image). Here, we use two RF voltage sources, namely *V*_ac1_ and *V*_ac2_ with amplitude of 250 mV. The connection between the two neuron layers is achieved by means of a resistive synaptic cross-bar array. We combine the concepts of bio-inspired unsupervised Spike-Timing Dependent Plasticity (STDP) (Bi and Poo, 1998) and astrocyte induced neural phase synchrony to automatically enable the network to learn to elicit such behavioral patterns, on the fly. The developed system sets off from an unlearnt state where all neurons have an independent response and remain unsynchronized in phase. However, upon system activation (and consequently astrocyte RF injection), the architecture eventually learns to bind the different possible configurations for the visual scene through phase correlation to either *V*_ac1_ or *V*_ac2_. It is to be noted that neurons *N*_1_, *N*_2_, *N*_3_, *N*_4_ comprise of pre-neurons while *N*_a_ and *N*_b_ are post neurons, separated by the resistive cross-bar array. Ultimately, a tight phase and frequency locking is observed among a particular pair of pre-neurons (*N*_1_, *N*_2_, and *N*_3_, *N*_4_) and post-neurons (*N*_*a*_ and *N*_*b*_). Due to random thermal fluctuations, the devices can converge to either of the two possible configurations for the bistable image, thereby illustrating the concept of optical illusion. The work can potentially pave the way for efficient hardware realization of coupled neuron-synapse-astrocyte networks enabled by compact neuromimetic devices.

### 4.3. Learning Phase Correlation

The premise for triggering the synchronous activity via astrocyte is accredited to the sensory attention as discussed before, and can be mapped in our proposed system to the amplitude of RF injection signal. Similar to better binding observed with increased attention, larger amplitudes lead to improved neural coupling. The strength of each input current to *N*_*a*_ and *N*_*b*_ is controlled by the synaptic conductances *G*_11_ − *G*_22_ of the memristive cross-bar array as shown in Figure 5B. Implementation of such cross-bar arrays with *in-situ* STDP learning has been previously explored for spintronic devices (Sengupta et al., 2016; Sengupta and Roy, 2017) and other post-CMOS technologies (Jo et al., 2010; Kuzum et al., 2011; Saha et al., 2021). It is worth mentioning here that each cross-connection also features a prior filtering “bias tee” to eliminate any possible DC current interactions among different devices. The DC paths of the bias tee are terminated to ground, while the AC signals get passed on to the cross-bar for coupling. Elaborating, the input AC current to the *j*_*th*_ post-neuronal device (considering HM resistance to be considerably lower in comparison to the synaptic resistances at each cross-point) can be described by Equation (5) as:


(5) $$I_{ac,N_{j}}(t)=\sum_{i}G_{ij}.V_{i}(t)$$

We now elucidate how our proposed architecture captures the essence of the optical illusion problem, shown in Figure 5, in reference frame of an observer. Specifically, the system should be able to adapt and converge to one of the possible interpretation discussed above. In particular, biologically inspired unsupervised STDP principles are used to train the programmable synaptic conductances (*G*_11_ − *G*_22_) in the cross-bar architecture for this purpose. The STDP weight (conductance) update equations are given by: Δ*w* = η_+_*w* exp($\frac{-\Delta t}{\tau_{+}}$) (for Δ*t* > 0) and Δ*w* = η_+_*w* exp($\frac{\Delta t}{\tau_{+}}$) (for Δ*t* < 0), where η_+_ and τ_+_ are learning hyperparameters, Δ*w* is the synaptic weight update and Δ*t* is the timing difference between the spikes corresponding to the selected post- and pre-neuron. The positive learning window (Δ*t* > 0) update occurs whenever a post-neuron fires while the negative learning window (Δ*t* < 0) update occurs at a pre-neuron firing event. It is worth pointing out here that we use a symmetric STDP learning rule in this work, i.e., the synaptic weight is potentiated for both the positive and negative learning windows. This is in contrast to the more popular asymmetric STDP observed in glutamatergic synapses (Bi and Poo, 1998), typically used in neuromorphic algorithms (Diehl and Cook, 2015). While symmetric STDP has also been observed in GABAergic synapses (Woodin et al., 2003), further neuroscience insights are required to substantiate the exact underlying mechanisms and cause of this plasticity. Asymmetric STDP is useful in application domains requiring temporal ordering of spikes, i.e., a pre-synaptic neuron spike will trigger a post-neuron spike. However, for our scenario, a temporal correlation is crucial irrespective of the sequence, which is enabled by the symmetric STDP behavior. Implementation of symmetric STDP in memristive cross-bar arrays can be easily achieved by proper waveform engineering of the programming voltage applied across the synapses (Serrano-Gotarredona et al., 2013; Sengupta et al., 2016). The cross-bar resistances are considered to have an ON/OFF resistance ratio of 10. The different input spike trains are derived from each device's magnetoresistance (MR) where a spike is triggered when the MR crosses its mean-value of 2 *KΩ*. Because *N*_1_ (*N*_3_) and *N*_2_ (*N*_4_) share a common HM, either of them can be used to extract the pre-neuron spikes during the weight update period. Besides STDP, a lateral inhibition effect (Diehl and Cook, 2015) is utilized. Whenever a spike occurs for any pre-neuron (post-neuron), the corresponding row (column) weights of the array are potentiated. However, the remaining rows (columns) are depressed proportionately. The lateral inhibition weight update equations are given by: Δ*w* = −η_−_*w* exp($\frac{-\Delta t}{\tau_{-}}$) (for Δ*t* > 0) and Δ*w* = −η_−_*w* exp($\frac{\Delta t}{\tau_{-}}$) (for Δ*t* < 0), where η_−_ and τ_−_ are learning hyperparameters, Δ*w* is the synaptic weight update and Δ*t* is the timing difference corresponding to the symmetric STDP weight update for the row or column which experiences weight potentiation. The lateral inhibition scheme is a simple extension of the synaptic programming voltage waveform engineering used in prior work (Indiveri et al., 2011; Serrano-Gotarredona et al., 2013; Sengupta et al., 2016). During the learning phase, this lateral inhibition effect causes the neuron under study to start responding selectively toward a specific configuration. This, in turn, enables the network to later converge to one of the interpretations for Figure 5A, as mentioned previously. The network simulation parameters are outlined in Table 2. The tabulated time-constants are measured with respect to the time-step for LLG simulation.

**Table 2:** Learning simulation parameters.

**Parameters**	**Value**
Time-step for LLG simulation	0.1 ps
STDP learning rate, η_+_	0.25
STDP time constant, τ_+_	5
Inhibition learning rate, η_−_	0.15
Inhibition time constant, τ_−_	5
Maximum synapse resistance in cross-bar array	25 *kΩ*

### 4.4. Simulation Results

**Figure 6:**
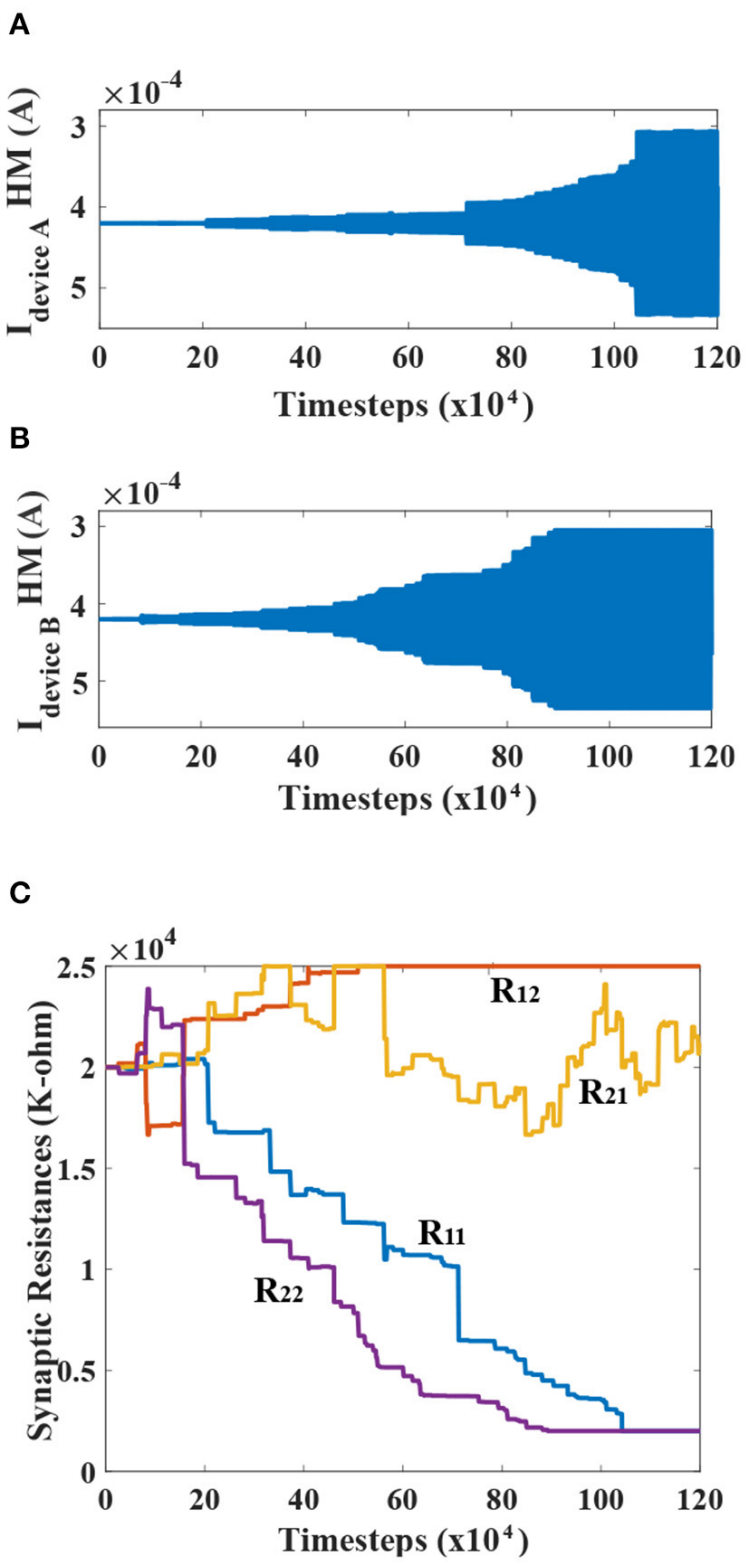
**(A,B)** The temporal evolution profiles of the net currents (DC+AC) flowing through the heavy metal for devices A and B are shown. The increasing AC amplitudes about the mean DC value can be seen. The relatively flattened envelopes post-learning suggest that the post-neuron devices are dominantly locked to one of the frequencies. **(C)** Temporal evolution of the cross-bar resistances during the learning process is shown.

The net currents for devices A and B, evolving through time, is portrayed for one of the simulations in Figures 6A,B respectively. Meanwhile, the corresponding synaptic resistances for the network are plotted in Figure 6C to elucidate the learning process discussed previously. The learning phase for the simulation is plotted as a function of timestep of the LLG simulation of the MTJ devices (0.1 ps). Observing the temporal profiles, an interesting deduction can be formulated, confirming that the different post-neurons get dominantly locked to different injection frequencies. The two sinusoids, being initially out of phase and adding up in comparable amounts for post-neurons, result in very low net currents. But, as the learning progresses, it becomes clear that one of the frequency gets dominant for a particular post-neuron, and thus the envelope tends to flatten in the end. It is worth mentioning here that the synaptic learning simulation in this work was performed from an algorithmic standpoint in a technology agnostic fashion. Depending on the underlying synapse technology, prior proposals for peripheral design for STDP learning needs to be considered (Serrano-Gotarredona et al., 2013; Sengupta et al., 2016). Since the focus of this article is on the MTJ neural synchrony aspect, we did not consider any specific synaptic device programming delay constraint (which is reflected in the instantaneous state changes of the synaptic connection strengths in Figure 6C). In reality, from a system design perspective, we need to have interleaved synaptic device state update phases that do not interfere with the neuron oscillation behavior (for instance, through decoupled write-read phases of three-terminal synaptic devices; Sengupta et al., 2016). The convergence was also not affected with reduced programming resolution of the synaptic connections (4-bits), thereby indicating resiliency to quantization (Hu et al., 2021).

**Figure 7:**
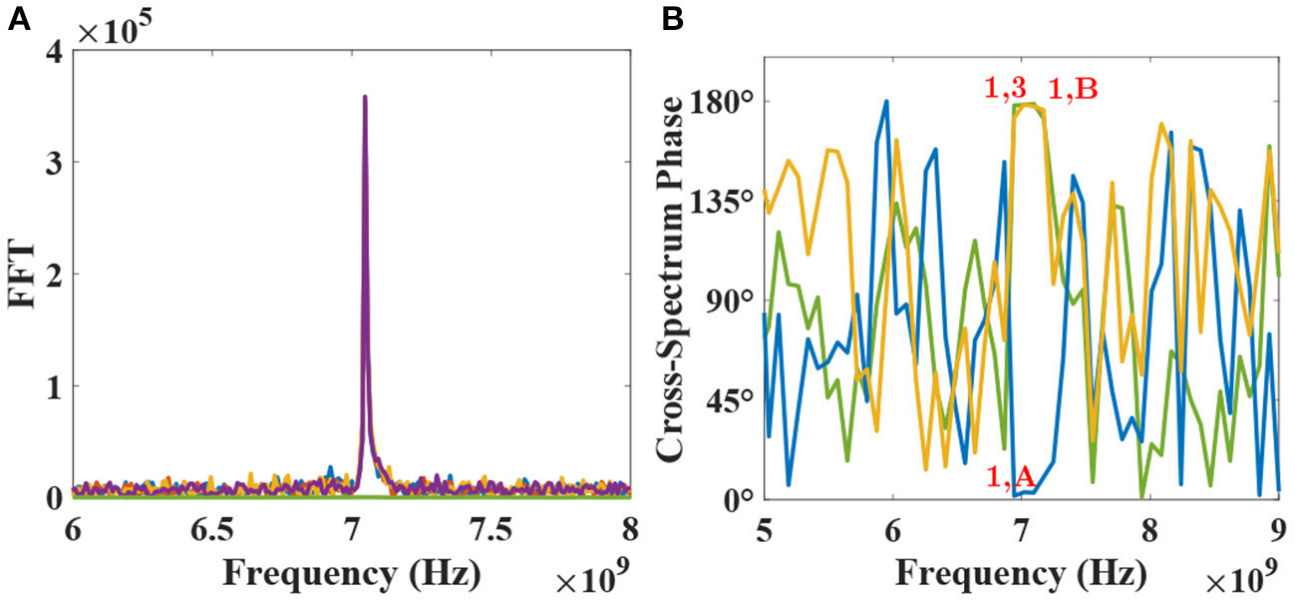
**(A)** FFT plots for all devices for one of the two possible configurations are shown post-learning. **(B)** Cross-spectrum phase for devices-pairs 1–3 (178.53°), 1-A (3.35°), and 1-B (178.3°) are plotted to show the phase-locking nature of the network post-learning at the injection frequency of 7.05 GHz.

Cohesing to one of the percept should surmise of a random event to provide equal chance for any of the two possible configurations to develop. Indeed, it is observed in our network that the synchronization occurs for random first and second layer neurons, post-training. Such a phenomenon can be accredited to the natural thermal fluctuations in our system, which tend to perturb the MTJ device's periodic nature. Figures 7A,B, respectively, depict the FFTs and cross-spectrum phase for various devices in the network for one such possible configuration upon learning termination. Specifically, cross-spectrum phases for device-pairs 1 & A (blue curve), 1 & 3 (yellow curve), and 1 & B (green curve) in Figure 7B are plotted to highlight that device 1, 2, and A get locked in phase at the injection frequency (7.05 GHz) while being completely out of phase with devices 3, 4, and B for the considered configuration.

**Figure 8:**
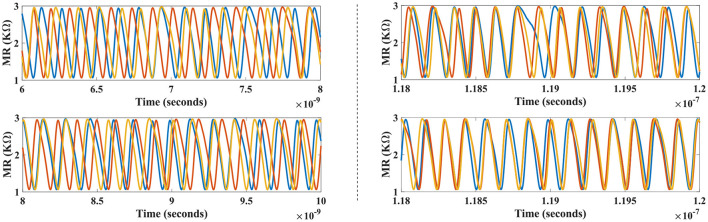
Temporal profile for the devices in the network (shown in Figure 5B) before **(left)** and after synchronization **(right)** are depicted for one particular configuration. Astrocyte functionality activates the synchronous regime causing learning to occur and subsequently coherent neural patterns are achieved for this configuration (a stochastic event). Devices *N*_1_, *N*_2_, and *N*_*a*_ (top-right panel) lock to injection signal with ϕ = 0°, while devices *N*_3_, *N*_4_, and *N*_*b*_ reveal concerted neural patterns in conjunction to ϕ = 180° injection signal (bottom-right panel).

Figure 8 plots the temporal profile of device magnetoresistance (MR) for *N*_1_, *N*_2_, and *N*_*a*_ devices in the top panel, along with MR of *N*_3_, *N*_4_, and *N*_*b*_ devices shown in the bottom panel. Initially all neuronal devices, albeit operating at the same free-running frequency (*f*_free_ = 7.05 GHz), elicit un-correlated phases, and hence temporal spike response due to devices' inherent thermal noise. After the astrocyte AC signal injection and STDP learning commences, it is observed that the devices *N*_1_ (*N*_3_) and *N*_2_ (*N*_4_) achieve a gradual coherent phase along with device *N*_*a*_ (*N*_*b*_), getting locked to the respective injection signal, as can be clearly seen in the right panels. The subsequent cross-correlation phase at the 7.05 GHz injection frequency post-synchronization averages to 1.6232° for the three-possible temporal profile pairs among *N*_1_, *N*_2_, and *N*_*a*_ (*N*_1_ ⋆ *N*_2_: 0.88°, *N*_2_ ⋆ *N*_*a*_: 2.136°, and *N*_1_ ⋆ *N*_*a*_: 1.856°). Likewise, *N*_3_, *N*_4_, and *N*_*b*_ after learning, achieve an average cross-phase of 1.848°. Bio-physically equivalent, this can be interpreted as a tight correlation among the attributes 1, 2, and *A*, corresponding to one of the interpretations of the bistable image. Finally, an increasing phase-mismatch is visible in neuronal outputs of all devices if the synchronization is revoked by the astrocyte, and the devices revert to their uncorrelated original free running frequency. This can be attributed to a diverted attention toward the sensory modal-input features leading to the impairment in correlated activity.

## 5. Discussion

Even though this work proves to be a good preliminary framework for emulating such brain-like functions, more investigation is required for decoding the neural code in such processes along with integrating these insights in Artificial Intelligence (AI) systems. For instance, selectivity bias toward some features among the myriad available sensory information, and, reductionism (down-streaming) of such higher-level modal inputs to local neuronal groups in the hierarchical structure, is poorly understood. There have been some efforts to study such processes using a reverse approach, where robots like Darwin VIII, inspired by the re-entrant neuroanatomy and synaptic plasticity, are developed and trained on visual mode data (Seth et al., 2004). In agreement with our work, they show synchronous activity binds different representative features of the detected object. Incorporating such connections in our system can be explored to further bridge the gap between real cortical networks and the respective inspired models. Supported by both neuroscience research and AI hardware developments, coupled astrocyte-neuron network architectures can potentially pave the way for a new generation of artificial cognitive-intelligence.

## Data Availability Statement

The original contributions presented in the study are included in the article/Supplementary Material, further inquiries can be directed to the corresponding author/s.

## Author Contributions

All authors contributed equally to the writing of the paper, developing the concepts, and performing the simulations.

## Funding

The work was supported in part by the National Science Foundation grant nos. BCS #2031632, ECCS #2028213, and CCF #1955815.

## Conflict of Interest

The authors declare that the research was conducted in the absence of any commercial or financial relationships that could be construed as a potential conflict of interest.

## Publisher's Note

All claims expressed in this article are solely those of the authors and do not necessarily represent those of their affiliated organizations, or those of the publisher, the editors and the reviewers. Any product that may be evaluated in this article, or claim that may be made by its manufacturer, is not guaranteed or endorsed by the publisher.
