# Supplementary material for: Emulation of Astrocyte Induced Neural Phase Synchrony in Spin-Orbit Torque Oscillator Neurons
Source: Front Neurosci. 2021 Oct 12;15:699632. doi: 10.3389/fnins.2021.699632 (PMC8546188; doi:10.3389/fnins.2021.699632)
Supplement: Supplementary file 1 [file Data_Sheet_1.pdf]

Emulation of Astrocyte Induced Neural Phase Synchrony in Spin-Orbit Torque Oscillator Neurons

Umang Garg[1,2], Kezhou Yang[1], and Abhronil Sengupta[1,*]

[1]School of Electrical Engineering and Computer Science, Department of Materials Science and Engineering, The Pennsylvania State University, University Park, PA, USA
[2]Department of Electronics and Instrumentation Engineering, Birla Institute of Technology and Science, Pilani, India
[*]sengupta@psu.edu

SUPPLEMENTARY INFORMATION

In order to better understand and substantiate the phase synchrony of the spin-torque oscillators, we considered the phase resetting curve (PRC) of the devices. The PRC plots the relation between phase shift and the timing of perturbation that causes this phase shift and describes the phase sensitivity of nonlinear oscillators to external perturbations[1]. There are two types of PRCs. Type-I PRC is non-negative or non-positive curve while type-II PRC has both negative and positive components in the curve[1].

To obtain the PRC through numerical calculations, we compared two oscillators. One of the oscillators was free-running with only a DC current pulse while the other perturbed oscillator was subjected to a pulse current applied in addition to the DC current, as is shown in Fig. 1(a). The phase shift due to the perturbation, $\Delta\phi$, is obtained from $\Delta\phi_i = 2\pi\Delta t_i/T_{period}$, where T_{period} is the oscillation period, $\Delta t_i = t_{f,i} - t_{p,i}$ is the i-th phase shift during the period (as shown in Fig. 1(b)). $\Delta\phi_i$ converges to a constant value after a period of time, which gives the value of $\Delta\phi$. ϕ is the phase of oscillator when perturbation is applied. The relation between $\Delta\phi$ and ϕ is the PRC of oscillator, which is plotted in Fig. 1(c). For our simulations, we considered $I_{DC} = 400\mu A$ and $I_{pulse} = 4\mu A$. Pulse width, T_{pulse}, is taken to be 20% of the oscillator period. The PRC shown in the figure is a type-II PRC. Oscillators with type-II PRC can be synchronized more efficiently under common noisy input compared to oscillators with type-I PRC, as is indicated in prior work[2].

To further understand the synchronization phenomena of a network of oscillators, we considered the Kuramoto model[3]. Coupling phenomena of spintronic oscillators for different schemes have been mapped to the Kuramoto model in prior works[4,5]. A phase sensitivity function augmented Kuramoto modelling approach has also been adopted to explain synchronization of spintronic oscillators under the application of pulse currents[6]. The Kuramoto model dynamics is described by,

$$\dot{\theta}_i = \omega_i + \sum_{j \neq i} K_{ij} \sin(\theta_j - \theta_i) \tag{1}$$

where, $\dot{\theta}_i$ is the actual frequency of the i-th oscillator, ω_i is the intrinsic frequency of the i-th oscillator, K_{ij} is the coupling strength between the i-th and j-th oscillator, and θ_i is the phase of the i-th oscillator.

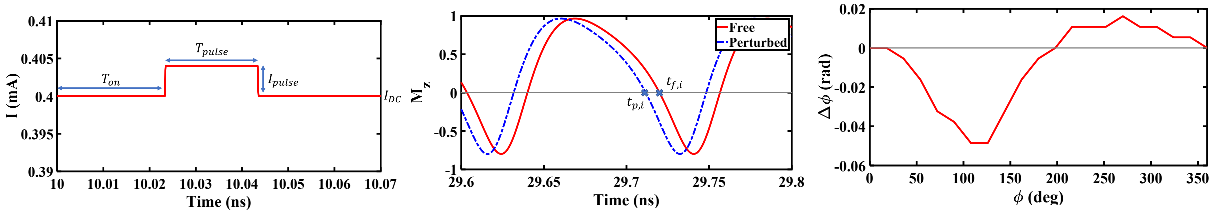

(a) (b) (c)

Figure 1. (a) Current pulse applied to the perturbed oscillator. T_{on} is the time instant when the current pulse is applied, which disregards the first 10 ns (since the oscillator reaches stable oscillation in 10 ns). T_{period} is the pulse width and I_{pulse} is the current amplitude. (b) Magnetization component in easy axis of the free and perturbed oscillators. The i-th time-instant when the free (perturbed) oscillator's magnetization reaches 0 is noted as $t_{f,i}$ ($t_{p,i}$). Phase shift $\Delta\phi_i$ is determined by $2\pi(t_{f,i} - t_{p,i})/T_{period}$, where T_{period} is the oscillation period. (c) PRC of the oscillator at $I_{DC} = 400\mu A$. Pulse amplitude is $I_{pulse} = 4\mu A$ and pulse width is $T_{pulse} = 0.02ns$.

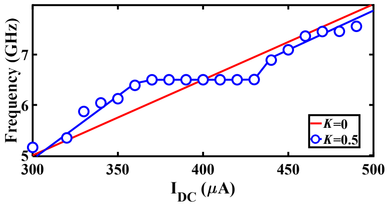

Figure 2. Kuramoto model oscillator coupling characteristics for non-zero coupling strength closely resembles the spin-oscillator synchronization characteristics. The AC injection frequency is $6.5GHz$.

In the coupling scheme shown in Fig. 3 of main text, multiple neuron devices are mounted upon a heavy metal layer through which an AC signal is driven by an astrocyte device. This AC signal provides the coupling signal between the driver device and each target neuron device. On the other hand, there is no signal from the neuron devices to the driver astrocyte device or between the target neuron devices (due to negligible dipolar coupling). Denoting the driver device by index 0 and the target neuron devices as $i > 0$, the coupling strength K_{ij} for the i-th target oscillator is given by,

$$K_{ij} = \begin{cases} 0 & j > 0 \\ f(I_{AC}) & j = 0 \end{cases} \tag{2}$$

where, I_{AC} is the amplitude of the AC signal. The DC bias current, I_{DC} of the oscillators control the intrinsic frequency ω_i. In this case, for the driver astrocyte device, the actual frequency $\dot{\theta}_0 = \omega_0$, and for the target neuron devices, $\dot{\theta}_i = \omega_i + K_{i0}\sin(\theta_0 - \theta_i)$, where $i > 0$. We consider a particular simulation setup of the Kuramoto model assuming a linear $\omega - I_{DC}$ relationship of the oscillators, similar to the one shown in Fig. 4(a) of the main text. As shown in Fig. 2, the Kuramoto model oscillator coupling characteristics bear close resemblance to the spintronic oscillator coupling characteristics in Fig. 4(a) of the main text, where the target oscillator actual frequency locks in to the driver frequency for a non-zero coupling strength.

References

1. Arai, H. & Imamura, H. Stochastic phase synchronization of perpendicularly magnetized spin-torque oscillators with the second-order uniaxial anisotropy. *IEEE Transactions on Magn.* **53**, 1–5, DOI: 10.1109/TMAG.2017.2704658 (2017).
2. Abouzeid, A. & Ermentrout, B. Type-ii phase resetting curve is optimal for stochastic synchrony. *Phys. Rev. E* **80**, 011911, DOI: 10.1103/PhysRevE.80.011911 (2009).
3. Acebrón, J. A., Bonilla, L. L., Vicente, C. J. P., Ritort, F. & Spigler, R. The kuramoto model: A simple paradigm for synchronization phenomena. *Rev. modern physics* **77**, 137 (2005).
4. Flovik, V., MacIà, F. & Wahlström, E. Describing synchronization and topological excitations in arrays of magnetic spin torque oscillators through the Kuramoto model. *Sci. Reports* **6**, 1–10 (2016).
5. Kabir, M. & Stan, M. *Synchronized Spin Torque Nano-Oscillators: From Theory to Applications*, 231–250 (Springer International Publishing, Cham, 2015).
6. Nakada, K. & Miura, K. Pulse-coupled spin torque nano oscillators with dynamic synapses for neuromorphic computing. In *2016 IEEE 16th International Conference on Nanotechnology (IEEE-NANO)*, 397–400 (IEEE, 2016).
